# Transfusion of Red Blood Cells Is Associated With Improved Central Venous Oxygen Saturation But Not Mortality in Septic Shock Patients

**DOI:** 10.14740/jocmr1843w

**Published:** 2014-09-09

**Authors:** Farid Sadaka, Steven Trottier, David Tannehill, Paige L Donnelly, Mia T Griffin, Zerihun Bunaye, Jacklyn O’Brien, Matthew Korobey, Rekha Lakshmanan

**Affiliations:** a Mercy Hospital St Louis; St Louis University, St. Louis, MO, USA

**Keywords:** RBC, Transfusion, Septic shock, EGDT, Early goal-directed therapy, Mortality, ScvO_2_, Central venous oxygen saturation

## Abstract

**Background:**

Although the optimum hemoglobin (H) concentration for patients with septic shock (SS) has not been specifically investigated, current guidelines suggest that H of 7 - 9 g/dL, compared with 10 - 12 g/dL, was not associated with increased mortality in critically ill adults. This contrasts with early goal-directed resuscitation protocols that use a target hematocrit of 30% in patients with low central venous oxygen saturation (ScvO_2_) during the first 6 hours of resuscitation of SS.

**Methods:**

Data elements were prospectively collected on all patients with SS patients (lactic acid (LA) > 4 mmol/L, or hypotension). Out of a total of 396 SS patients, 46 patients received red blood cell (RBC) transfusion for ScvO_2_ < 70% (RBC group). We then matched 71 SS patients that did not receive RBC transfusion (NRBC group) on the following goals (G): LA obtained within 6 hours (G1), antibiotics given within 3 hours (G2), 20 mL/kg fluid bolus followed by vasopressors (VP) if needed to keep mean arterial pressure > 65 mm Hg (G3), central venous pressure > 8 mm Hg within 6 hours (G4) and ScvO_2_ > 70% within 6 hours (G5).

**Results:**

In the RBC group, after one unit of RBC transfusion, ScvO_2_ improved from average of 63% (± 12%) to 68% (± 10%) (P = 0.02). Sixteen patients required another unit of RBC, and this resulted in increase of ScvO_2_ to 78% (± 11%) (P < 0.01). The RBC and NRBC groups were matched on sequential organ failure assessment (SOFA) scores and all five goals. There was no difference in mortality between the two groups: 41% vs. 39.4% (OR: 0.8, 95% CI: 0.4 - 1.7, P = 0.6).

**Conclusions:**

In our study, transfusion of RBC was not associated with decreased mortality in SS patients.

## Introduction

In the United States, approximately 750,000 cases of sepsis occur each year, of which at least 225,000 are fatal. If it also causes organ dysfunction, the diagnosis is severe sepsis. If severe sepsis is accompanied with tissue hypoperfusion, the diagnosis is septic shock (SS). Organ failure occurs in about one-third of patients with sepsis, and severe sepsis is associated with an estimated mortality rate of 30-50%. There is wide variation in the incidence of sepsis and severe sepsis in the intensive care unit (ICU) setting, with reported rates ranging from 20% to 80%, and reported mortality of 20% to 50%. SS, defined as a state of acute circulatory failure characterized by persistent hypotension unexplained by other causes, despite adequate fluid resuscitation, affects between 10% and 30% of patients managed in the ICU, and its incidence is increasing. Mortality from SS in the ICU is estimated to range between 45% and 63% in observational studies [1-7].

Guidelines published as part of the surviving sepsis campaign (SSC) [8] have endorsed use of red blood cells (RBCs) in the treatment of patients with severe sepsis and SS who show evidence of hypoperfusion. This recommendation is primarily based on studies that evaluated a bundle approach to patients in severe sepsis [9]. RBC transfusion to obtain a hematocrit of 30% is included in this bundle for patients with a central venous oxygen saturation (ScvO_2_) < 70%. Patients achieving this goal had better outcomes than patients who did not reach the goal. The specific effect of transfusion was not evaluated in this study, however, as the investigation was designed to assess the overall bundle rather than its component parts. On the other hand, several studies, summarized in literature [10] point to several problems documented with RBC transfusions, such as infection, pulmonary complications such as transfusion-related acute lung injury, fluid overload, transfusion-related immunomodulation, multiorgan failure and increased mortality. As a result, outside this window during the first 6 h of early-goal directed therapy (EGDT) for severe sepsis and SS, a “restrictive” strategy of RBC transfusion (transfuse when Hb < 7 g/dL) is recommended.

The primary objective of this study is to evaluate the effect of RBC transfusion on ScvO_2_ and mortality when used during EGDT for patients with SS.

## Methods

As discussed above, following SSC guidelines with rapid recognition and aggressive intervention of SS dramatically improves outcome. As a result, a sepsis team has been created in our institution in order to increase compliance with SSC guidelines. As soon as an SS patient is identified anywhere in the hospital, the sepsis team is called to go to the bedside and initiate EGDT. Data are prospectively collected on these patients in order to examine the effectiveness of this effort and its impact on improving compliance with the EGDT bundle and outcomes. As a result, data elements were prospectively collected on all patients. This study is a retrospective review of this database.

SS patients (defined as lactic acid (LA) > 4 mmol/L, or hypotension persistent after initial fluid challenge) admitted between June 2011 and March 2013 were included. We identified a total of 396 SS patients. The protocol was as follows: As soon as a patient was diagnosed with sepsis and sepsis team is notified, an LA was measured. Once patient is diagnosed with SS, a 20 mL/kg bolus of crystalloid was given to achieve a central venous pressure of 8 - 12 mm Hg. If the mean arterial pressure was less than 65 mm Hg, vasopressors were given to maintain a mean arterial pressure of at least 65 mm Hg. Once these goals are achieved, if the central venous oxygen saturation was less than 70%, RBCs were transfused to achieve a hematocrit of at least 30%.

Forty-six patients received RBC transfusion for ScvO_2_ < 70% (RBC group). Of the remaining patients, we then matched 71 SS patients that did not receive RBC transfusion (NRBC group) on the following goals (G) based on SSC 2008 criteria [11]: LA obtained within 6 h (G1), antibiotics given within 3 h (G2), 20 mL/kg fluid bolus followed by vasopressors (VP) if needed to keep mean arterial pressure (MAP) > 65 mm Hg (G3), central venous pressure (CVP) ≥ 8 mm Hg within 6 h (G4) and ScvO_2_ ≥ 70% within 6 h (G5). We calculated average age, average sequential organ failure assessment (SOFA) scores and fluid balance at the end of 6 h for both groups. In the RBC group, we collected ScvO_2_ levels before and after each unit of RBC transfused. Outcome was hospital mortality. Matching is used to evaluate the effect of a treatment by comparing the treated and the non-treated patients in an observational study (especially when the treatment is not randomly assigned). The goal of matching is for every treated patient, to find one non-treated patient (in this case transfusion versus no transfusion) with similar observable characteristics against whom the effect of treatment can be assessed. By matching treated patients to similar non-treated patients, matching enables a comparison of outcomes in order to estimate the effect of the treatment without reduced bias due to confounding. In this case, all of the goals (G1-G5) could confound outcome and thus were all matched. The RBC and NRBC groups were compared by Pearson Chi-squared and Fisher’s exact tests to analyze statistical significance. Mean, standard deviation and P value were reported for each comparison. Statistical significance was defined as P ≤ 0.05. This study was approved by Mercy Hospital Institutional Review Board.

## Results

**Figure 1:**
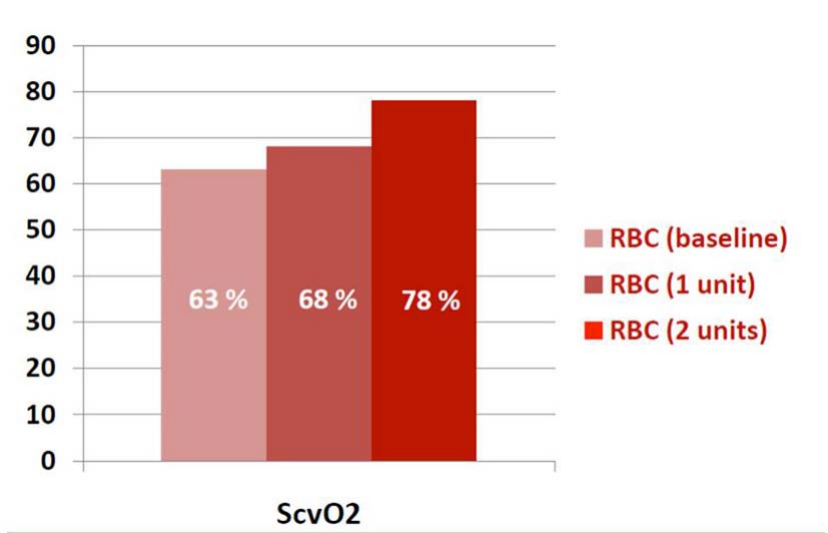
ScvO_2_ values after one and two units in the RBC group.

In the RBC group, after one unit of RBC transfusion, ScvO_2_ improved from average of 63% (± 12%) to 68% (± 10%) (P = 0.02). Sixteen patients required another unit of RBC, and this resulted in increase of ScvO_2_ to 78% (± 11%) (P < 0.01) (Fig. 1). All these transfusions were for ScvO_2_ < 70% as part of EGDT. None of the transfusions were for other indications, such as bleeding, hemolysis, or bone marrow dyscrasias. Although all patients in the RBC group got transfused, not all of them achieved this goal (ScvO_2_ ≥ 70%) at the 6 h mark, which explains the findings in Table 1.

**Table 1:** Characteristics of RBC and NRBC Groups With Septic Shock

	RBC	NRBC	P value
N	46	71	
Age, mean, SD	71 (15)	65 (17)	0.06
SOFA score, mean, SD	8.6 (3.9)	8.4 (3.4)	0.8
Fluid balance at 6 h, mL, mean, SD	3,500 (1,700)	4,000 (2,100)	0.2
Lactic acid	4.4 (± 3.9)	3.9 (± 2.8)	0.4
Vasopressors, n, %	29 (63%)	40 (56%)	0.5
LA within 6 h, n, % (G1)	43 (93%)	68 (96%)	0.6
Antibiotics with 3 h, n, % (G2)	31 (67%)	40 (56%)	0.2
20 mL/kg, then vasopressors, n, % (G3)	35 (76%)	57 (80%)	0.6
CVP ≥ 8 mm Hg within 6 h, n, % (G4)	21 (46%)	29 (41%)	0.5
ScvO_2_ ≥ 70% within 6 h, n, % (G5)	12 (26%)	19 (27%)	0.9

**Figure 2:**
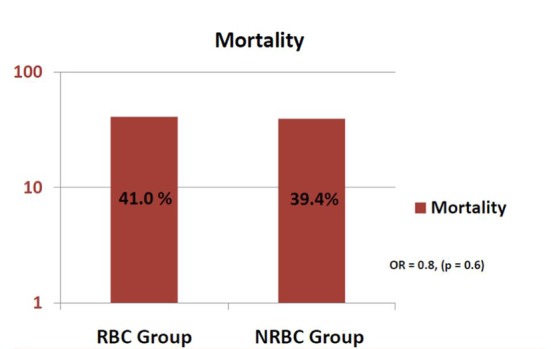
Hospital mortality of the RBC and NRBC groups.

The two groups were matched on age and severity of illness. For the RBC group and NRBC group respectively, age in years was 71 (± 15) vs. 65.9 (± 17) (P = 0.06), and SOFA score 8.6 (± 3.9) vs. 8.4 (± 3.4) (P = 0.8). The RBC and NRBC groups were also matched on goals of resuscitation as follows: fluid balance in mL at 6 h was 3,500 (± 1,700) vs. 4,000 (± 2,100) (P = 0.2). LA was 4.4 (± 3.9) vs. 3.9 (± 2.8) (P = 0.4), VPs were used in 29 (63%) vs. 40 patients (56%) (P = 0.5), the goal of obtaining LA within 6 h was achieved in 43 (93%) vs. 68 patients (96%) (P = 0.6), the goal of giving antibiotics within 3 h was achieved in 31 (67%) vs. 40 patients (56%) (P = 0.2), the goal of achieving MAP > 65 with fluid and VP was achieved in 35 (76%) vs. 57 patients (80%) (P = 0.6), CVP goal was achieved within 6 h in 21 (46%) vs. 29 patients (41%) (P = 0.5) and ScvO_2_ goal was achieved within 6 h in 12 (26%) vs. 19 patients (27%) (P = 0.9) (Table 1). There was no difference in mortality between the two groups: 41% vs. 39.4% (OR: 0.8; 95% CI: 0.4 - 1.7, P = 0.6) (Fig. 2).

## Discussion

RBC transfusion is one of the most commonly used interventions in the ICU to treat severe anemia, which often occurs in SS. In the United States, more than 14 million units of RBCs are administered annually, many of which are administered in the ICU and in SS patients [12]. Approximately 40-80% of RBC transfusions are not given for bleeding, but rather for low hemoglobin levels, for a decrease in physiological reserve, or for alterations in tissue perfusion [13, 14]. Forty to fifty percent of septic and other critically ill patients require blood transfusion during their ICU stay [15]. By day two in the ICU, nearly 95% of patients are anemic [13].

For years, it was considered that a hemoglobin concentration of 10 g/dL, or a hematocrit of 30%, represented the lowest level acceptable, thereby providing a standard and convenient “transfusion trigger” [16]. This was adopted based on the fact that tissue oxygen delivery (DO_2_) is the product of tissue blood flow and arterial oxygen content. Tissue blood flow is thus determined by cardiac output and regional vasoregulation, and arterial oxygen content depends on hemoglobin concentration and its percentage saturation. Oxygen flow increases as the hemoglobin falls to a level termed the “optimal hematocrit”, at which point DO_2_ is highest at the lowest energy cost to the individual. This occurs around a hematocrit of 30% [17]. Below this “optimal” level, the maintenance of tissue oxygen consumption (VO_2_) and aerobic metabolism at decreasing levels of DO_2_ is principally provided by increased oxygen extraction. In the critical care environment, there have been several studies examining the interrelationship between hematocrit, DO_2_ and VO_2_. Shoemaker et al [18] and Boyd et al [19] initially defined the optimal hematocrit at around 30 % since, below this level, oxygen delivery and consumption were decreased in critically ill patients and mortality was increased. Above this level, there was no change in these variables or outcome. This line of reasoning led to the common practice of maintaining this “10/30 rule” as the transfusion trigger. Subsequently, several problems were documented with RBC transfusions, such as infection, pulmonary complications such as TRALI and transfusion-associated circulatory overload (TACO), transfusion-related immunomodulation (TRIM) and multiorgan failure, and increased mortality [20]. This lead to the need for evaluation of restrictive transfusion strategies.

The best evidence available regarding the efficacy of RBC transfusion among critically ill patients including SS is from a randomized controlled trial, the transfusion requirements in critical care (TRICC) trial, conducted by the Canadian Critical Care Trials Group [21]. In this study, a liberal transfusion strategy (hemoglobin 10 - 12 g/dL, with a transfusion trigger of 10 g/dL) was compared to a restrictive transfusion strategy (hemoglobin 7 - 9 g/dL, with a transfusion trigger of 7 g/dL) in a general medical and surgical critical care population. Patients who were euvolemic after initial treatment who had a hemoglobin concentration < 9 g/dL within 72 h were enrolled. The TRICC trial documented an overall nonsignificant trend toward decreased 30-day mortality in the restrictive group; however, there was a significant decrease in mortality in the restrictive group among patients who were less acutely ill (APACHE II scores < 20) and among younger patients (< 55 years of age). Patients in the restrictive group received 54% less RBC units than those in the liberal group [21]. The diversity of patients enrolled in the trial and the consistency of the results suggest that the conclusions may be generalized to most critical care patients including SS patients. In a recent analysis by the Cochrane database of 19 trials involving a total of 6,264 patients, restrictive transfusion strategies were associated with a statistically significant reduction in hospital mortality (RR: 0.77, 95% CI: 0.62 - 0.95) but not 30-day mortality (RR: 0.85, 95% CI: 0.70 - 1.03) [22]. The authors concluded that the existing evidence supports the use of restrictive transfusion triggers in most patients including those with pre-existing cardiovascular disease.

Guidelines published as part of the SSC [8] have endorsed use of RBCs in the treatment of patients with severe sepsis and SS who show evidence of hypoperfusion. EGDT thus calls for an increase in hematocrit to at least 30% in the setting of perceived oxygen deficit. The specific effect of transfusion was not evaluated in this study, however, as the investigation was designed to assess the overall bundle rather than its component parts. The pathologic effects of RBC transfusion in sepsis could be numerous. Several studies have demonstrated that RBC rheology is impaired (increased aggregation, decreased deformability, alterations of RBC shape) in recipient RBCs in SS patients [23-26]. RBC can also act as oxygen sensor, which can modulate tissue oxygen flow variables, by the release of the vasodilators, nitric oxide [27, 28] or ATP [29]. This release of vasodilators from RBCs during hypoxia could be impaired during storage and/or sepsis/septic shock. Storage of RBCs decreases levels of 2,3-diphosphoglycerate and adenosine triphosphate (ATP) levels with a resultant increase in oxygen affinity and a decrease in the ability of hemoglobin to offload oxygen. Morphological changes in erythrocytes occur during storage which may result in increased fragility, decreased viability and decreased deformability of RBCs. A release of a number of substances occurs during storage resulting in such adverse systemic responses as fever, cellular injury, alterations in regional and global blood flow, and organ dysfunction. Using near infrared spectroscopy (NIRS) or sidestream dark field (SDF), several investigators have reported that microcirculation is markedly altered in sepsis, that these alterations are more severe in nonsurvivors than in survivors, that persistent microvascular alterations are associated with development of multiple organ failure and death and that microvascular alterations are the most sensitive and specific predictor of outcome in septic patients [30-34]. In severe septic patient requiring leukoreduced RBC transfusion, using sidestream dark field (SDF), Sakr et al showed that the sublingual microcirculation was globally unaltered; however, it improved in patients with altered capillary perfusion at baseline [35]. Using both SDF and NIRS, Sadaka et al looked at patients that got non-leukoreduced RBCs for a hemoglobin < 7.0, or for a hemoglobin between 7.0 and 9.0 with either lactic acidosis or central venous oxygen saturation < 70% [36]. Sadaka et al showed that muscle tissue oxygen consumption, microvascular reactivity and sublingual microcirculation were globally unaltered by RBC transfusion in severe septic and SS patients. However, muscle oxygen consumption improved in patients with low baseline and deteriorated in patients with preserved baseline [36]. In this study, we showed that transfusion of RBC during the EGDT period of resuscitation of SS was not associated with decreased mortality, despite improvement of ScvO_2_ values. Improvement in oxygen delivery with RBC transfusion could have been offset by the multiple pathophysiologic effects of transfusing RBCs. Given the clinical evidence and the aforementioned pathophysiologic alterations associated with RBCs, our findings are thus not surprising and further question the rationale of transfusion during EGDT for SS patients.

Our study has several limitations. The small sample size is an obvious limitation. Although we prospectively collected robust data and accounted for bundle compliance data during EGDT, we still cannot exclude the possibility of unaccounted, unmatched or missing data, which may lead to bias and undetected differences in baseline characteristics. In addition, there may be unaccounted differences in treatment of the patients in the two groups, which can lead to differences in outcomes. Although the two groups were matched on basic characteristics and goals of EGDT, exact matching remains challenging due to the retrospective nature of the study, the small number of patients, and the inability to match on baseline hematocrit and ScvO_2_ levels which lead to one group (RBC group) getting transfused and not the other group (NRBC group). However, patients are closely matched on severity of illness as apparent by nonsignificant difference in SOFA scores between the two groups. Another limitation is lack of multivariate analysis that was not used due to the small number of patients. By the same token, a power analysis was not undertaken since this is a retrospective review of data and thus the results of this study are considered hypothesis generating and not conclusive. All this makes drawing final conclusions difficult based on findings of this trial alone.

### Conclusion

In our study, transfusion of RBC was not associated with decreased mortality in SS patients, despite improvement in ScvO_2_ values. This and other studies mentioned above raise a concern about liberal transfusion during resuscitation phase of SS patients. This study does suggest that better means of identifying a need for transfusion are needed and that blindly transfusing to an arbitrarily set (and high) Hb may be detrimental. Future research with larger samples is needed to further examine the association between RBC transfusion and outcomes of patients resuscitated early in SS.
